# Immune modulation in malignant pleural effusion: from microenvironment to therapeutic implications

**DOI:** 10.1186/s12935-024-03211-w

**Published:** 2024-03-12

**Authors:** Shan Ge, Yuwei Zhao, Jun Liang, Zhongning He, Kai Li, Guanghui Zhang, Baojin Hua, Honggang Zheng, Qiujun Guo, Runzhi Qi, Zhan Shi

**Affiliations:** 1 Institute of Basic Research in Clinical Medicine, China Academy of Chinese Medical Sciences, No. 16, Nanxiao Street, Dongzhimen, Dongcheng District, Beijing, 100700 China; 2 Department of Oncology, Guang’anmen Hospital, China Academy of Chinese Medical Sciences, No. 5 Beixiange, Xicheng District, Beijing, 100053 China; 3 Guang’anmen Hospital, China Academy of Chinese Medical Sciences, No. 5 Beixiange, Xicheng District, Beijing, 100053 China; 4 Beijing Shijitan Hospital, No.10 Yangfangdiantieyilu, Haidian District, Beijing, 100038 China; 5 Beijing University of Chinese Medicine, Chaoyang District, Beijing, 100029 China

**Keywords:** Malignant pleural effusion, Immunotherapy, Immune microenvironment, Immune cells

## Abstract

Immune microenvironment and immunotherapy have become the focus and frontier of tumor research, and the immune checkpoint inhibitors has provided novel strategies for tumor treatment. Malignant pleural effusion (MPE) is a common end-stage manifestation of lung cancer, malignant pleural mesothelioma and other thoracic malignancies, which is invasive and often accompanied by poor prognosis, affecting the quality of life of affected patients. Currently, clinical therapy for MPE is limited to pleural puncture, pleural fixation, catheter drainage, and other palliative therapies. Immunization is a new direction for rehabilitation and treatment of MPE. The effusion caused by cancer cells establishes its own immune microenvironment during its formation. Immune cells, cytokines, signal pathways of microenvironment affect the MPE progress and prognosis of patients. The interaction between them have been proved. The relevant studies were obtained through a systematic search of PubMed database according to keywords search method. Then through screening and sorting and reading full-text, 300 literatures were screened out. Exclude irrelevant and poor quality articles, 238 literatures were cited in the references. In this study, the mechanism of immune microenvironment affecting malignant pleural effusion was discussed from the perspectives of adaptive immune cells, innate immune cells, cytokines and molecular targets. Meanwhile, this study focused on the clinical value of microenvironmental components in the immunotherapy and prognosis of malignant pleural effusion.

## Introduction

Malignant pleural effusion (MPE) is a common complication of advanced malignant tumours that mainly results from the pleural metastasis of malignant tumours (such as lung cancer and breast cancer) and pleural mesothelioma. MPE progresses rapidly, severely restricts lung expansion, and affects cardiopulmonary function, often leading to respiratory distress and circulatory disturbances that severely affect patient survival. The current clinical local treatment for MPE is intrathoracic chemotherapy, which not only causes extensive fibrosis and adhesions in the pleura but can also lead to multiple chemotherapy drug resistance in some patients. Therefore, it is critical that new treatment methods that can prolong survival and improve quality of life are found. Among the many treatment options available, immunotherapy has emerged as a promising strategy that can provide clinical benefit to cancer patients. The tumor microenvironment (TME) in MPE results from malignant tumour modification, and together with the tumour, it determines the progress of MPE development and the prognosis of the disease. The TME includes several types of immune cells, tumour cells, stromal cells, the extracellular matrix, and many soluble molecules [1]. TME maturation, which is induced by autocrine and paracrine communication between the different TME cells, leads to an increase in the cellular release of inflammatory factors, increased angiogenesis, and enhanced vascular permeability, leading to an increase in the inflammatory exudation and hemorrhagic components within the MPE [2]. In addition, TME cells can also reduce immune activation signals and down-regulate the recognition and presentation of antigens through immune cell metabolic reorganization [3], leading to immunosuppressive effects in the MPE environment [4] and promoting the growth and immune escape of floating tumour cells in the MPE. A variety of immune cells, such as T lymphocyte subsets, tumour-associated macrophages (TAMs), dendritic cells (DCs), natural killer (NK) cells, myeloid-derived suppressor cells (MDSCs), and B lymphocytes in the MPE-TME are immunosuppressed by the MPE, allowing unrestrained growth of the tumour cells that escape from immune surveillance in the MPE. Without the vascular support of the primary tumour, malignant cells in the MPE do not respond to conventional anti-tumour drugs, and it is therefore important that more effective immunotherapies are developed. Current studies into programmed cell death receptor-1 (PD-1)- or programmed cell death ligand-1 (PD-L1)-mediated immunity have indicated that PD-L1 expression in MPE tumour cells is significantly correlated with PD-L1 expression in macrophages, suggesting interaction between tumour cells and MPE macrophages [5]. The progression-free survival (PFS) and overall survival (OS) of patients with MPE have been found to be significantly shorter than those without MPE [6]. Garcia et al. [7] found that tumour effector CD8^+^ T cells were susceptible to negative regulation by PD-L1 when not fully differentiated into effector cells, and that the PD-L1/PD-1 pathway could inhibit the function of tumour effector T cells in the MPE of lung cancer patients. However, the mechanism and clinical significance of the higher PD-L1 expression in MPE have not been fully elucidated and more experimental studies and prospective clinical trials are thus required. In addition to PD-1/PD-L1, increasing numbers of immune checkpoints, targets, signaling pathways, cytokines that play immune regulatory roles in the microenvironment, and non-coding RNAs that are delivered via exosomes have been discovered, enabling new breakthroughs in MPE immunotherapy. This paper describes the formation mechanism of the TME in MPE and discusses the current research into immunotherapy.

## Methods

**Fig. 1:**
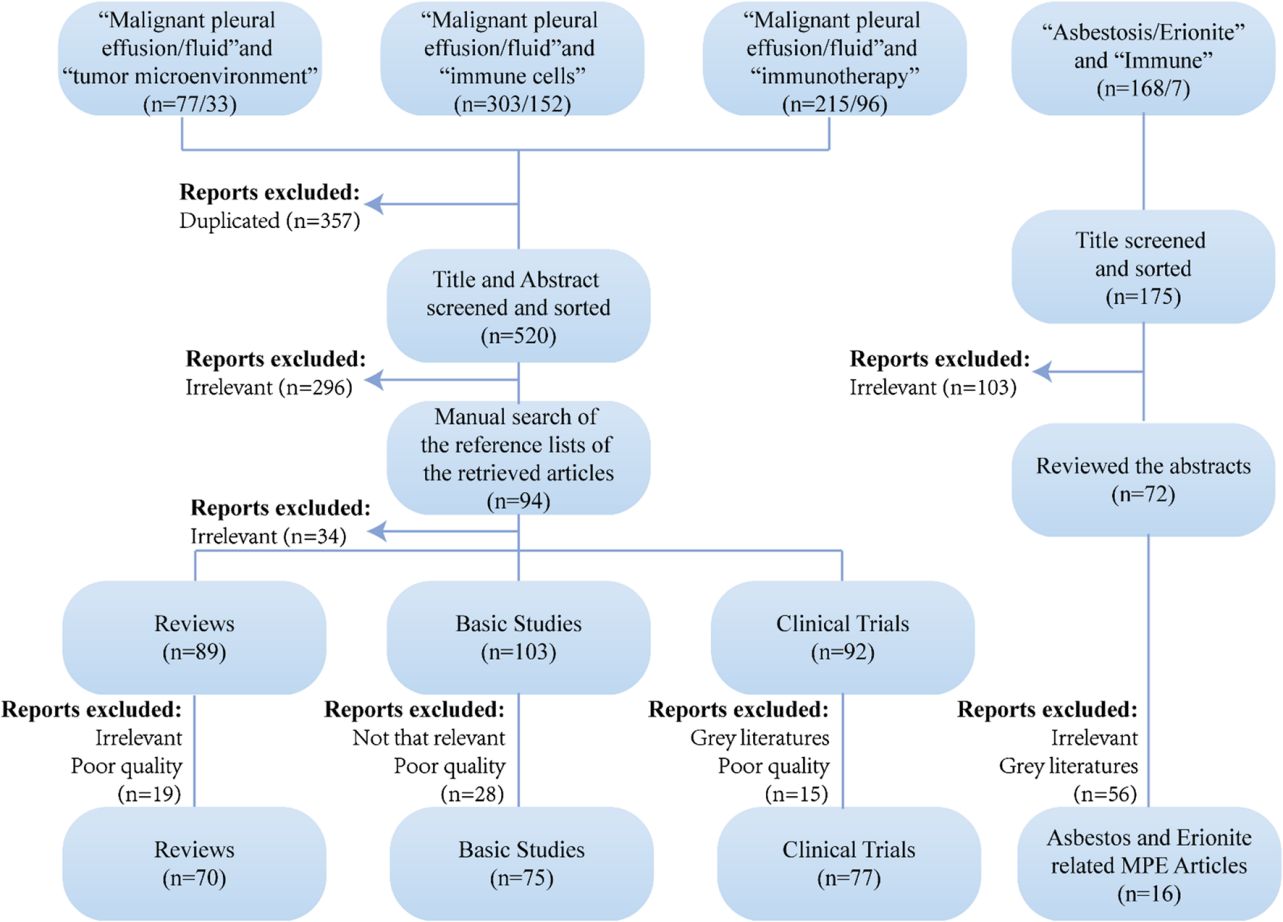
Flow chart of the literature screening process. MPE, malignant pleural effusions

Studies limited to English language were identified by searching PubMed and supplemented by a manual search of the references of the papers retrieved. A preliminary search with the keyword “malignant pleural effusion/malignant pleural fluid” in all fields gives more than 8000 or 5000 results. Therefore, we conducted a post hoc search of PubMed to reduce the results, adding words included “malignant pleural effusion/malignant pleural fluid and tumor microenvironment” or “malignant pleural effusion/malignant pleural fluid and immune cells” or “malignant pleural effusion/malignant pleural fluid and immunotherapy” to the search strategy. After screening articles’ titles and abstracts for eligibility, 224 articles were considered for full-text analysis. We downloaded the relevant papers and further screened to identify potentially eligible studies. Then we conducted a manual search of the reference lists of the retrieved articles yielded a total of 94 articles, from these, 60 articles were screened for eligible articles. The selected studies were further sorted into three categories: reviews, basic research including cell studies, animal studies and clinical sample studies, and clinical trials. We search with the keyword “asbestosis/erionite and immune” as well. Then we excluded 159 articles that were not relevant articles on the basis of their titles and abstracts. A total of 16 asbestos-related articles are included in the review. And we screened the final remaining articles to make sure that these studies are relevant with immunity mechanism and immunotherapy of MPE, and estimated the eligibility for inclusion of the full text (Fig. 1).

## Adaptive immune cells in MPE-TME

### T lymphocytes

MPE is a common complication of malignant tumours. In immunocompromised patients, tumour cells can achieve immune escape through a series of mechanisms, and cellular immunity dominates in the clearance of tumour cells. T lymphocytes are the main components in MPE immune cells, which mainly include Helper T (Th) Cells, cytotoxic T cells (CTLs), and regulatory T cells (Tregs).

#### Th cells

CD4^+^ T lymphocytes dominate the Th cell population and the T cell receptors (TCR) in Th cells are involved in the recognition of antigens. Th1 cells are differentiated under the direction of IL-12 and mainly secrete interleukin-2 (IL-2), interferon-γ (IFN-γ), and tumour necrosis factor-β (TNF-β) to inhibit tumour cell growth and promote the cellular immune response. Ghayumi et al. [8] found that the level of IFN-γ, a Th1 cell cytokine, was lower in the MPE that results from lung cancer than that associated with extrathoracic tumours, suggesting that the number of Th1 cytokines produced is closely related to the site of the tumour that is resulting in the production of MPE. Th1 cells are known to inhibit the development of tumours by eliciting an inflammatory response. IL-2 can promote the Th1 cell response, enhance the cytotoxic activity of CTLs, and boost cellular immune function. Chen et al. [9] used a combination of IL-2 and IL-12 to stimulate MPE lymphocytes *in vitro* and promote the secretion of IFN-γ with results indicating that the Th2 was converted to Th1 and the anti-tumour activity of cells was restored. Wu et al. [10] demonstrated that IL-10 can promote MPE and accelerate the death of MPE-bearing mice by suppressing the differentiation of T cells into Th1 cells and the blocking chemokine receptor 3 (CXCR3)/chemokine ligand 10 (CXCL10) signaling pathway that recruits Th1 and Th17 cells into the thoracic cavity. Another study indicated that IL-10 significantly increases the mRNA and protein expression levels of G protein-coupled receptor 55 (GPR55) via miR-7116-5p. In addition, GPR55 was found to inhibit the proliferation of Th1 cells via the extracellular regulatory protein kinase (ERK) signaling pathway, exacerbating MPE production [11].

In the presence of IL-4, CD4^+^ T cells differentiate into Th2 cells, which mainly secrete cytokines such as IL-4, IL-5, and IL-10, enhancing the humoral immune response [12]. Oshikawa et al. [13] found that significantly higher concentrations of IL-4 and IL-10 were secreted by the Th2 cells in the MPE, with IL-12 below the minimum detectable level. Later experiments involving the isolation of T cells in order to compare the concentrations of cytokines indicated that Th2 cells predominate the lymphocytes in MPE. Wu et al. demonstrated that IL-10 can promote the occurrence of MPE and accelerate the death of MPE-bearing mice by suppressing the differentiation of T cells into Th1 cells and blocking the chemokine receptor 3 (CXCR3)/chemokine ligand 10 (CXCL10) signaling pathway that recruits Th1 and Th17 cells into the thoracic cavity. The concentrations of both CXCL9 and CXCL10 were significantly higher in IL-10^/^ mice with MPE than in wild-type (WT) mice, and knockdown of the CXCL10 gene in the tumour cells led to a decrease in the recruitment of Th1 and Th17 cells into the MPE, increasing both the volume of the MPE and the mortality of the MPE-bearing mice; however, the mechanism that led to this occurrence has not yet been confirmed in human clinical studies. Dysfunctional Th1/Th2 cells in MPE may impact the pathophysiological process of pleural disease, leading to a drift of cellular immunity toward Th2 and resulting in increased immunosuppression and poor patient prognosis.

Th17 cells also play an important role in MPE, with the differentiation of these cells from the initial CD4^+^ T cells induced by IL-6 and TGF-β. The main cytokines that are secreted by Th17 cells include IL-17, IL-21, and IL-23 [14]. IL-21 and TGF-β together stimulate the differentiation of CD4^+^ T cells into Th17 cells, expanding the Th17 cell population, and IL-23 matures and stabilizes Th17 cells to maintain their function [15]. Ye et al. [16] showed that the number of Th17 cells in MPE was significantly higher than that in peripheral blood, and alterations to the proportions of these cells could predict the risk of death and the degree of survival in lung cancer patients with MPE. The IL-6, TGF-β1, and IL-1β concentrations were higher in the MPE than the serum. Moreover, chemokine ligand 20 (CCL20) and chemokine ligand 20 (CCL22) were able to induce the aggregation of Th17 cells in MPE, producing higher concentrations of these cells in the MPE than the serum [17]. The expression of CCR6 and CCR4 via the CCL20 and CCL22 receptors, respectively, were found to be highly expressed in Th17 cells in both the blood and MPE [16]. Studies investigating the use of immunotherapy against CCL20 and CCL22, have shown that anti-CCL20 and anti-CCL22 monoclonal antibodies can impair the chemotaxis of Th17 cells, both alone or in combination, and that the inhibitory effect is stronger when they are combined [16]. Lu et al. [18] confirmed that IL-17 deficiency promotes the neovascularization and angiogenesis of pleural tumours, promoting the formation of MPE. IL-17 promotes the differentiation of Th9 cells by up-regulating the expression of interferon regulatory factor 4 (IRF4) and GATA-3, accelerating the release of IL-9, recruiting more inflammatory cells to the site of tumour cells, and activating the immune response of the CD8^+^ T lymphocytes [19], thus inhibiting Tregs functionality and reducing MPE formation and mortality in MPE-bearing mice. In addition, IL-17 deficiency can also promote neovascularization and angiogenesis in pleural tumours and the pleural vascular permeability, facilitating the formation of MPE and accelerating death in MPE-bearing mice [20]. The expression of IL-26 in Th17 cells can up-regulate IL-22 secretion by promoting the proliferation and differentiation of Th22 cells, and high levels of the IL-26 protein indicate a poor prognosis, suggesting that IL-26 may be an important cytokine affecting the pathogenesis of MPE [21].

Th9 cells can also affect the progress of MPE by regulating IL-9, and it has recently been discovered that Th9 cells can secrete IL-9 with multifunctional activity, unlike Th1, Th2, and Th17 cells [22, 23]. One study indicated that increased numbers of Th9 cells in the MPE of patients with lung cancer suggests shorter survival periods for patients with lung cancer [24]. Ye et al. [24] observed that IL-9 and Tregs can promote the number of Th9 cells in the MPE. IL-9 can significantly promote the proliferation of tumour cells and prevent IFN-γ-induced tumour cell apoptosis by activating the signal transducer and activator in the transcription 3 (STAT3) signal pathway. One study [25] showed that pleural mesothelial cells may recruit Th9 cells into MPE by producing CCL20 and that Th9 cells express low levels of CCR7, meaning that they can easily migrate into the thoracic cavity of patients with MPE. IL-9 released by Th9 cells induces the differentiation of Th17 cells and activates Tregs functioning through the STAT3 and STAT5 signaling pathways [26], which in turn promote IL-17 release and inhibit MPE formation.

The polarization of Th22 cells from naive CD4^+^ T cells is mainly stimulated by TNF-α, IL-6, and IL-1β [27]. Research indicates that IL-22, the signature cytokine of Th22 cells, is involved in the occurrence and development of tumours [28]. Zhang et al. [29] found that the high expression of IL-22 in the MPE, primary tumour tissues, and serum of patients with non-small cell lung cancer (NSCLC) may be related to the initiation and progression of the disease. A study by Ye et al. [30] showed that the upregulation of Th22 cells and IL-22 in MPE may play an important role in the immunoregulation of cancer cells in human MPE, and that Th22 cells may be recruited into the MPE via the CCL20-CCR6, CCL22-CCR4, and/or CCL27-CCR10 axes. IL-26 is another crucial cytokine in Th22 cells. Niu et al. [21] found that IL-26 can lead to the proliferation of CD4^+^IL-22^+^ T cells rather than CD4^+^IL-17A^+^ T cells, indicating that IL-26 can selectively up-regulate the number of Th22 cells produced. The data also show that IL-26 has a strong positive effect on CD4^+^IL-22^+^ T cells and may directly promote the differentiation of memory Th22 cells, probably via TNF-α and IL-6. At present, the mechanism by which MPE production is inhibited via Th22 remains obscure, suggesting promising directions for future research.

##### CTL cells

Cytotoxic T lymphocytes (CTLs) are considered to be the main lymphocyte subsets that kill cancer cells. CTLs can specifically recognize tumour cells via the chemokines secreted by DCs and the signaling pathway of CD4^+^ T cells, and they secrete perforin and granzyme B to induce apoptosis in target cells [31, 32]. CTLs can also mediate programmed cell death through the Fas pathway in a Ca^2+^-deficient environment or in the absence of perforin and granzyme [33, 34]. CD3^+^CD8^+^ T cells are the predominant CTLs in MPE.

CD8^+^ T lymphocytes have a direct apoptosis effect and can recognize tumour antigens that are represented by major histocompatibility complex (MHC) class I molecules on the surface of tumour cells and directly lyse tumour cells. CD8^+^ T cells play an important role in host anti-tumour immune responses and are associated with a better prognosis for various tumours. CD8^+^ T cells that were isolated from MPE by Dhupar et al. [35] remained cytotoxic and produced sth 24 h after *in vitro* culture. The experiment was continued using CD8^+^ T cells that were co-cultured with CD45^-^ tumour containing cells (non-hematopoietic MPE cells) or autologous peripheral blood mononuclear cells at 1:1 for 24 h. Tumor cell lysis and IFN-γ production were found to significantly increase in the co-cultured cells, suggesting that a subset of the MPE-derived CD8^+^ T cells responded to autologous tumour-containing target cells in vitro [35]. However, multiple studies have shown that the function of CD8^+^ T cells is gradually inhibited in the TME, suggesting that the immune function of exhausted CD8^+^ T cells (TEX) in the MPE can be reversed, rendering TEX a possible focus for future research [36, 37]. Studies have found that tumour-specific CD8^+^ T cells appear highly dysfunctional and exhibit an exhausted phenotype, which contributes to tumour immune evasion and metastasis. TEX cells are associated with a decrease in the secretion of cytokines such as IL-2, granzyme B (GzmB), and interferon (IFN)-γ, which is accompanied by decreased cell viability and proliferation and increased apoptosis [38, 39]. Hu et al. [40] demonstrated that the CD8^+^ T cells in lung cancer patients with MPE were functionally impaired, with an exhausted phenotype. Several studies have confirmed that PD-1 expression is 1.58 times higher in MPE CD8^+^ T cells than it is in peripheral blood. PD-1 activation inhibits the activation and proliferation of T lymphocytes and the production of cytokines (including interferon-γ and TNF-α) [41, 42], which results in dysfunctional CD8^+^ T cells. However, IL-2 treatment reduced the PD-1 expression, reversed MPE CD8^+^ T cell exhaustion and promoted the generation of GzmB and IFN-γ [40]. These findings suggest that the targeted control of T cell exhaustion through anti-PD-1 and IL-2 therapy may be an effective therapeutic strategy for MPE in the future. T cell immunoglobulin mucin-3 (TIM-3) has also been found to be highly expressed in exhausted CD8^+^ T cells, and blocking TIM-3 signaling *in vivo* was found to reverse CD8^+^ T cell exhaustion [43, 44]. The positive expression of TIM-3 in CD8^+^ T cells was detected in the MPE of patients with malignant pleural mesothelioma (MPM) [45]. TIM-3^+^PD-1^+^CD8^+^ T cells exhibited more severe deficiency than TIM-3^-^PD-1^+^ cells in their ability to produce IFN-γ, TNF-α, and IL-2 [44]. Therefore, combining anti-TIM-3 antibodies with anti-PD-1 therapies to reverse the function of CD8^+^ T cells may restore the immune response and provide novel targeted MPE therapies. Cytotoxic T lymphocyte-associated protein-4 (CTLA-4) has been found able to bind to TEX cells and blocking CTLA-4 with antibodies has been shown to enhance the anti-tumour immune response in mouse preclinical models [46]. The proportion of CTLA-4^+^ cells in CD4^+^ T cells and CD8^+^ T cells has been found significantly higher in MPE than peripheral blood in lung cancer patients [47]. MPE control is also thought possible by targeting CTLA-4; however, no relevant studies have as yet been conducted investigating this possibility. CTLA-4 and PD-1 have both been shown to attenuate T cell activation by different mechanisms [48]^.^ PD-1 blockade mainly induces the expansion of exhausted CD8^+^ T cells, while CTLA-4 blockade induces the expansion of inducible costimulatory molecule positive (ICOS^+^) Th1-like CD4^+^ effector T cells as well as CD8^+^ T cells [49]. The co-involvement of these two distinct cellular mechanisms has led to the clinical observation that combining anti-CTLA-4 with anti-PD-1 therapy enhances anti-tumour efficacy [50]. This also suggests that combining PD-1, TIM-3, and CTLA-4 may be highly significant in MPE-targeting therapies. Chemokines, cytokines, and other soluble factors that are secreted by tumour cells or stromal cells increase the sensitivity of CD8^+^ T cells to instigate activation-induced cell death (AICD) by inducing the amplification of non-human leukocyte antigen (non-HLA) restrictive inflammatory responses [36]. MPE CD8^+^ T cells were observed to undergo AICD; however, this phenomenon was not found in peripheral blood [51]. The sensitivity of MPE CD8^+^ T cells to AICD has been associated with upregulation of the apoptosis-related factor ligand (FasL) and expression of the tumour necrosis factor-related apoptosis-inducing ligand (TRAIL) [51]. Therefore, AICD is involved in killing target cells via the action of CD8^+^ T cells in the pleural cavity, inhibiting cellular immunity. Caspase-8/9 inhibitors rescue CD8^+^ T cells from AICD; additionally, blocking the Fas/FasL pathway can prevent CD8^+^ T cells from being affected by AICD and reduce MPE formation [51]. IL-26 may be able to impair cytotoxicity in CD8^+^ T cells [21]. The expression level of granzyme B was significantly lower in IL-26-stimulated CD8^+^ T cells than in unstimulated CD8^+^ T cells, suggesting sth. Furthermore, the number of MDSCs correlated with the activation potential of CD8^+^ T cells, and MDSC-related immunosuppression was observed after the CD8^+^ T cells were removed from the MPE, suggesting that MDSCs or factors associated with an MDSC-enriched environment may lead to durable T-cell dysfunction via epigenetic effects [35]. It is apparent that the bone marrow-derived granulocyte or monocyte populations in the MPE may also inhibit the killing of tumour cells by CD8^+^ T cells, which is also one of the important reasons that MPE aggravation occurs.

Another subset of T cells in the MPE, CD3^+^CD4^+^ T lymphocytes, can activate the antigenic immune response state by secreting cytokines, with their function regulated by immune cytokines. CD4^+^ T cells mainly mediate anti-tumour immunity by facilitating CD8^+^ CTL and antibody responses, by expressing key molecules that are associated with cytolytic granules such as granzymes or perforin or via direct cytotoxicity to tumour cells [52, 53]. Effective anti-tumour immunity depends on CD4^+^ T cells, and CD4^+^ T cells acquire their specificity for autologous tumours through the cross-activation of tumour antigens by autologous APCs [54]. A specific subset of CD4^+^ T cells has been observed in the peripheral circulation of NSCLC patients [55]. Laheurte et al. found that higher levels of telomerase reverse transcriptase (TERT)-specific Th1 type CD4^+^ T cells in the peripheral blood of NSCLC patients were associated with better prognosis [56]. At present, no report has been made on the relationship between CD4^+^ CTL and MPE; however, it has been acknowledged that CD4^+^ T cells can improve anti-tumour immunity. Whether this can be applied to the MPE in the future deserves attention.

##### Treg cells

Tregs are a subgroup of inhibitory T cells that play an immunosuppressive role following activation in the body via the secretion of IL-4, IL-10, and TGF-β and participate in the immune escape mechanism of tumours, promoting the occurrence and development of tumours [57]. Chen et al. [58] observed more Tregs in the MPE of lung cancer patients than in the peripheral blood of those without MPE, indicating that Tregs may be involved in the local immune response. Several studies have also reported that the CD4^+^/CD25(high)/FoxP3^+^/CD127^-^ Tregs content is related to the prognosis of lung cancer patients, and the number of Tregs in the bronchoalveolar lavage fluid of patients with lung cancer has been found to be higher than that of patients with benign lesions [59]. Other studies have reported that higher levels of Tregs indicate more advanced tumours and lower survival rates [60, 61]. Delong et al. [62] found that the CD4^+^CD25^+^ Tregs content in the MPE associated with various cancers also differed. Evaluation of the CD45^+^CD3^+^CD4^+^CD25^+^ T cells in MPE by flow cytometry indicated proportions of CD25^+^ T cells in small cell lung and breast cancers, respectively, which is significantly higher than that associated with pleural mesothelioma. These results suggest that by detecting the number and distribution of Tregs, the local immune microenvironment and immunosuppression status of MPE patients can be analysed, which is conducive to the development of immunotherapy against Tregs targets. The expression of miR-141 in tumour cells decreases and the production of CXCL1 increases during MPE development, and miR-141 recruits Tregs into MPE via CXCR2, resulting in an enhanced immunosuppressive effect of Tregs that promotes the immune escape of tumour cells and exacerbates MPE formation. The miR-141-CXCL1-CXCR2 signal transduction pathway may thus be an important potential factor that reduces the survival rate of patients [60]. The level of CCL22 in the MPE is significantly higher than that of serum, and classical pleural CCL22 has been found to be mainly produced by macrophages, T cells, and malignant tumour cells [63]. However, Tregs were found to strongly express CCR4, CCL17, or CCL22 chemokine receptor on their surfaces in the peripheral blood of MPE patients, suggesting that CCL22 may be related to Tregs aggregation in MPE [17]. CCL22 can recruit a large number of Tregs to exert an inhibitory effect, and Tregs further recruit relevant immune cells with immunosuppressive functions, forming a vicious cycle and blocking the anti-tumour effect of the immune system [64]. Another study found that [65] both CD39^+^ Tregs and Th17 cells increase in MPE, and the number of CD39^+^ Tregs is negatively correlated with Th17 cells. CD39^+^ Tregs inhibit the generation and differentiation of pleural Th17 cells through a latency associated peptide (LAP)-dependent mechanism that promotes the formation of MPE. Yu et al. [66] found that significant accumulations of CD4^+^CD25^+^FOXP3^+^ Tregs in the MPE contained high levels of Helios which would further increase the level of CD4^+^CD25^+^FOXP3^+^ Tregs [67]. It has been reported that an increase in the rORγt^+^Foxp3^+^ Tregs levels of the MPE in NSCLC patients suggests a new Treg phenotype. In addition, the expression level of CD4^+^CD25^+^Foxp3^+^ Tregs has also been positively correlated with the expression level of IRF4 in MPE. These findings suggest that IRF4 may potentially promote the transformation of MPE Tregs to Th17-like phenotypes by regulating Helios. At present, IL-8 has mainly been found in the Foxp3^+^ Tregs [68]. A study by Zarogoulids et al. [69] reported that IL-8 enhanced matrix metalloproteinase-2 and -9 activity, increasing the metastatic activity of underlying malignancies. IL-8 is known to induce angiogenesis by activating vascular endothelial growth factor, and has been suggested another important reason for the formation of MPE. A recent study found that the higher the frequency of the TNFR2^+^ T regulatory proteins in the MPE, the more tumour cells that are present and the greater the amount of MPE. This suggests that TNFR2^+^ Tregs may promote the invasion and metastasis of tumour cells into the pleural cavity, promoting the occurrence and development of MPE [70].

### B lymphocytes

The anti-tumour role of B cells in the TME has received less attention, but its role in the TME should not be ignored. B cells promote tumour progression by promoting angiogenesis, generating pro-inflammatory responses, and directly or indirectly inhibiting T cell activation [71–73]. However, B cells also play a dual role in tumour immunity [74], with their known anti-tumour activity, the ability to directly kill tumour cells, functionality as antigen-presenting cells, and ability to recognize antigens and facilitate the production of specific antibodies. Wu et al. [75] showed that B cells promote the formation of MPE *in vivo* by regulating CD4^+^ T cells. B cells were significantly decreased in the MPE as compared to the blood and spleen in LLC and MC38206 mouse models. The absolute counts of B cells and naive B cells in were significantly lower in the MPE than the peripheral blood of patients, and naive B cells comprised the majority of those observed in the MPE [75]. Activated naive B cells can inhibit the proliferation of Th17 cells through the PD-1 and PD-L1 pathways; however, the mechanism by which these cells promote the proliferation of Th1 cells remains to be elucidated [75]. However, PD-L1 mAb therapy can reverse Th17 expansion. Thus, naive B cells contribute to the formation of MPE by modulating the Th1/Th17 cell response, tilting conditions towards anti-tumour cell immunity [75]. The expression of B7-H4, which is associated with a poor prognosis in patients with metastatic pleural adenocarcinoma, is also elevated in B cells. The intrapleural injection of anti-B7-H4 mAb in MPE mouse models has been shown to effectively inhibit the formation of MPE [76]. Future research should thus focus on an in-depth exploration of the immune regulation that is associated with B cells and expand the list of known MPE immune cells.

## Innate immune cells in the MPE-TME

### Tumour-associated macrophages (TAMs)

TAMs are important components in the immune microenvironment and can be divided into M1-like and M2-like types. M1-like TAMs secrete pro-inflammatory cytokines such as IL-1, IL-6, IL-12, and TNF-α, along with reactive oxygen species and nitrogen, which are essential for host defense and tumour cell death [77]. M2-like TAMs release anti-inflammatory cytokines such as IL-10, prostaglandin E2, TGF-β, matrix metalloproteinases (MMPs) and VEGF, inhibiting the immune surveillance of tumours, promoting tumour growth and metastasis, and participating in angiogenesis, tissue repair, remodeling and other processes [78]. Narayanan et al. [79] found that M1 polarized macrophages can improve the survival rate of patients, indicating that M1 polarized macrophages have an anti-tumour effect [80]. In contrast, TAMs are polarized to the M2-like phenotype by cytokines (such as IL-10, M-CSF, or TGF-β) that are secreted by tumour cells. TNF-α and IL-10, which are secreted by M2-like macrophages, can induce PD-L1 expression in an autocrine manner, and inhibiting T cell function, which has a strong promoting effect on the occurrence and development of tumours [81]. Studies have indicated that CD163^+^CD14^+^ TAMs can be used as reliable diagnostic markers for MPE. Flow cytometry-based detection of the frequency of CD163^+^CD14^+^ TAMs in pleural effusion samples had a sensitivity of 81.2 % and a specificity of 100 %. Moreover, patients with low levels of CD163^+^CD14^+^ TAMs showed higher survival rates and better prognosis, indicating that CD163^+^CD14^+^ TAMs can be used as a potential prognostic biomarker for MPE [82]. Multiple studies have reported that TGF-β can inhibit the function of effector T cells [83, 84]. Li et al. found that the elevated expression level of TGF-β in MPE resulted mainly from TAMs, meaning that blocking the release of TGF-β may reverse the cytotoxic effects of CD4^+^ and CD8^+^ T cells in MPE. Wang et al. [68] found that the amount of CCL22 produced by MPE-derived TAMs (mainly CD163^+^ TAMs) was significantly higher than that of MPE-derived Tregs. Treg-derived IL-8 induced the upregulation of TGF-β in TAMs, which further mediated the CCL22 production by TAMs, promoting the formation of an immunosuppressive TME in the MPE.

### Dendritic cells (DCs)

DCs are the most powerful antigen-presenting cells in the human body, and can efficiently ingest, process, and present tumour antigens that participate in the immune response. Immature DCs have strong antigen uptake and migration capabilities, while mature DCs can activate naive T cells, stimulate T cell proliferation [85], and affect the progression of various tumour diseases. DCs are generally divided into conventional DCs (cDCs), plasmacytoid DCs (pDCs), and monocyte-derived DCs (MoDCs), which play various roles in tumour immunity. cDCs are divided into two types, cDC1 and cDC2. The induction of CD8^+^ T cell activity by cDC1 via cross-presentation plays a key role in tumour rejection and immunotherapy [86] and is also critical for the early initiation of CD4^+^ T cells [87]. cDC2 cells mainly stimulate the proliferation of CD4^+^ T cells, thereby participating in the anti-tumour immune response [88]. pDC-secreted type I interferon promotes anti-tumour immunity, and the function of MoDC depends on factors in the TME [89]. Elevated cytokines (such as IL-10 and VEGF) have also been found to inhibit the function of DCs in MPE [89]. DCs in the MPE may be immunosuppressed, and it is difficult to exert effective antigen presentation. For the first time, Gu et al. [90] identified a new immature DC subset, CD16^-^BDCA1^+^ cells (infDCs), in the MPE of patients with NSCLC, which showed strong phagocytic activity at both 37 ℃ and 4 ℃. InfDCs can induce the differentiation of autologous memory CD4^+^ T cells into Th1 cells upon activation by TLR 4, 7, and 8 agonists (LPS and R 848). However, the immunosuppressive state of MPE in patients with lung cancer means that it is difficult to induce the InfDC anti-tumour immune response *in vivo*. In general, the function and mechanism of DCs in MPE is still poorly understood.

### Myeloid-derived suppressor cells (MDSCs)

MDSCs are a population of immature myeloid cells that are derived from bone marrow and include two main subsets; polymorphonuclear MDSCs (PMN-MDSCs) and monocytic MDSCs (M-MDSCs). MDSCs are immunosuppressive cells that are mainly composed of immature macrophages, DCs, and granulocytes. MDSCs promote tumour angiogenesis by producing vascular endothelial growth factor (VEGF) and facilitate tumour cell invasion and metastasis by producing MMPs [91]. In addition, MDSCs can facilitate the formation of the pre-metastatic niche (PMN) in tumour cells, thereby promoting the development of tumours [92]. The increased expression level of VEGF in the pleural effusion of MPE patients has been considered an important marker with significant diagnostic value [93]. VEGF promotes the occurrence and development of MPE through two integrated mechanisms; increasing the vascular permeability (direct effect) and promoting angiogenesis (indirect effect) [94]. It is apparent that the enhanced production of VEGF is one of the mechanisms that aggravates MPE formation by MDSCs. MDSCs can evade immune surveillance and attack via multiple pathways during tumour development. In TME, MDSCs enhance their own production of IL-10 [95, 96] and IL-12 by down-regulating the production of IL-12 by macrophages. Decreased levels of IL-12 inhibit the killing activity of NK cells towards tumour cells [97] and induce and recruit Tregs [98], while high levels of IL-10 interfere with DC maturation. The interaction between MDSCs and macrophages polarizes M1 macrophages to the M2 phenotype, enhances M2 macrophages, suppresses both the adaptive and the innate anti-tumour immunity, and promotes the growth of tumour cells [95, 96]. MDSCs form an inhibitory immune microenvironment in the MPE through the above mechanisms, promoting the development of malignant cells in the MPE. MDSCs inhibit the activation of CD4^+^ and CD8^+^ T cells by inhibiting cysteine uptake by T cells and down-regulating L-selectin in T cells [96]. Studies have demonstrated that the targeted depletion of MDSCs can induce and enhance the killing ability of CTLs towards tumour cells, proving that the failure of immune surveillance that is associated with cancer may be partially attributed to MDSCs [99]. The level of MDSCs in lung adenocarcinoma-derived MPE has been found to be significantly increased [100], indicating that MDSCs play an inhibitory role in the immune microenvironment of MPE. However, the specific mechanism by which MDSCs act in the MPE has not as yet been reported upon in relevant literature.

### Natural killer cells (NK cells)

NK cells are a group of innate immune cells in the body that can kill target cells non-specifically, independent of antigen sensitization. They are not restricted by the MHC and play an important role in tumour immune surveillance. NK cells kill tumour cells by secreting perforin and granzymes or induce tumour cell apoptosis by interacting with the TNF-related apoptosis-inducing ligand (TRAIL) via death receptor signaling [101]. NK cells form the first line of defense against viruses and cancer because they can detect early signs of tumour metastasis and respond immediately [102]. However, tumour cells can evade NK cells via a variety of strategies, such as the secretion of cytokines such as TGF-β, IL-10, and prostaglandin E2 (PGE2) [103]. A study by Fend et al. [104] showed lower numbers of NK cells in the peripheral blood of NSCLC patients than normal people, suggesting that tumour cells can achieve immune escape by down-regulating NK cells. NK cell aggregation has also been found in MPE. Vacca et al. [105] found that NK cells in MPE (MPE-NK cells) are functionally active and can release cytokines; when activated by IL-2, the killing activity of MPE-NK cells against tumour cells was even higher than that of NK cells in the peripheral blood (PB-NK cells) activated by autologous IL-2. PE-NK cells have strong tumouricidal ability and are not substantially inhibited by the tumour pleural microenvironment [106]. As the inhibitory cytokines produced by NK cells may be diluted in the pleural effusion, the concentration may be inadequate to induce an inhibitory effect [105]. Numerous clinical trials have demonstrated that IL-2 has toxic effects and induces Tregs proliferation, which may compromise the anti-tumour response. IL-15, unlike IL-2, is not toxic. Croxatto et al. [107] continued Vacca’s research to prove that PE-NK cells that have been activated by IL-15 *in vitro* can control tumour growth in vivo; IL-15 not only overcomes the inhibitory effect of soluble factors in MPE, but also restores the effector function of PE-NK cells that have been damaged by exposure to autologous MPE. In the malignant pleural environment, PE-NK cells promote vascular endothelial cell proliferation and induce the growth of capillary-like structures via the production of VEGF [108]. VEGF can enhance vascular permeability [109] to become a key factor in the formation of MPE. Immune checkpoints TIM-3 and LAG-3 can affect anti-tumour responses by inhibiting lymphocyte activity; however, NK cells in the MPE of MPM patients showed high TIM-3 and LAG-3 expression [45], meaning that TIM-3 and LAG-3 are likely to provide new opportunities for targeted MPE therapies.

**Fig. 2:**
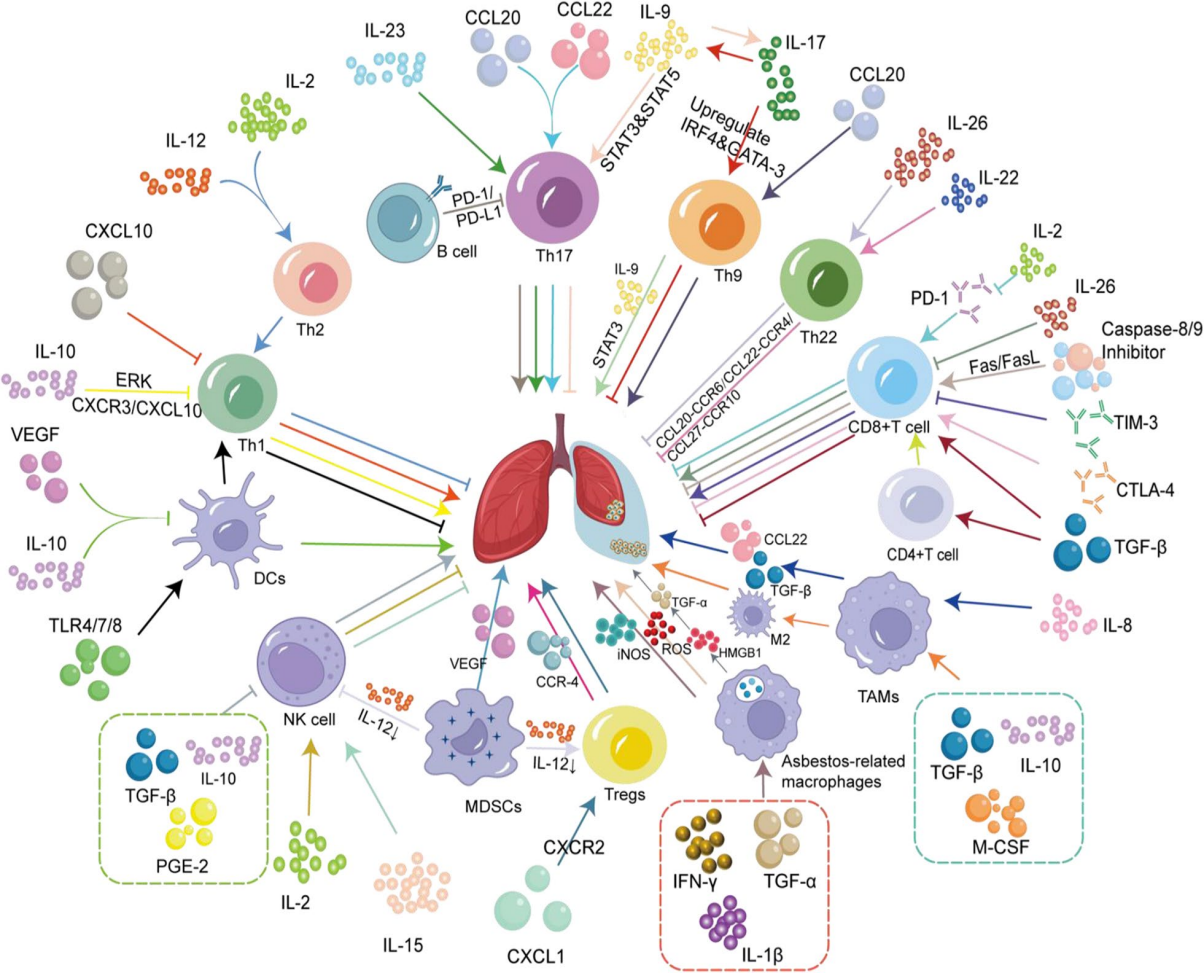
The interaction between immune cells, cytokines, and tumor cell-dominated MPE

The roles of various immune cells in the immune microenvironment of MPE have been summarized in Table 1. The interaction between immune cells, cytokines, and tumour cell-dominated MPE were shown in Figure 2.

**Table 1:** Effect of immune cells on MPE in TIME of lung cancer

Cell type		Other cytokines involved in regulation of MPE	Related cytokines involved in regulation of MPE	Mechanism of effect	Promote( +)/inhibit(−) the formation of MPE	References
Th cells	Th1	IL-2, IFN-γ, TNF-β	IL-12	Stimulate IFN-γ synthesis and polarize Th2 phenotype	–	[9]
		/	IL-10	Suppress Th1 differentiation, proliferation and Th1, Th17 recruitment. Enhance GPR55 mRNA and protein expression	+	[10, 11]
	Th2	IL-4, IL-5, IL-10	CXCL10	Th1 and Th17 low accumulation lead to CXCL10 reduce	+	[10]
	Th17	IL-17, IL-21, IL-23, IL-26	IL-23	Promote Th17 maturation	–	[15]
		/	IL-9	Drive Th17 differentiation, activate Tregs, induce IL-17 release	+	[26]
		/	IL-17	Enhance angiogenesis and proliferation of tumor cells, increase permeability of the pleural microvessels	+	[20]
		/	CCL20, CCL22	Induce recruitment of Th17 in MPE	+	[19]
		/	IL-26	Promote proliferation and differentiation of Th22, upregulate IL-22	–	[21]
	Th9	IL-9	IL-9	Promote proliferation of tumor cells	+	[24]
		/	CCL20	Induce recruitment of Th9 in MPE	+	[25]
		/	IL-17	Promote Th9 differentiation and activate the immune response	–	[18]
	Th22	IL-22, IL-26	IL-22	Promote the recruitment of Th22 to pleural cavity	–	[30]
		/	IL-26	Increase CD4^+^IL-22^+^T-cell subsets	–	[21]
CTLs	CD3^+^CD8^+^T cell	IL-2, Perforin, Granzyme B	IL-2	Decrease PD-1 expression and reverse CD8^+^T cells exhaustion	–	[40]
		/	TIM-3	Decrease IFN-γ, TNF-α and IL-2 production	+	[44]
		/	CTLA-4	Reduce ICOS^+^Th1-like CD4^+^ T cells and CD8^+^T cells	–	[49]
		/	Caspase-8/9 Inhibitor	Rescue CD8^+^T cells and block Fas/FasL signaling pathway	–	[51]
		/	IL-26	Gzm B and killing capacity of impair CD8^+^T cell was decreased	+	[21
	CD3^+^CD4^+^T cell	IFN-γ, TNF-α, IL-2	APCs	Specific CD4^+^T cell subsets generate autologous tumor-specific immune responses	–	[56]
Tregs		IL-4, IL-10, TGF-β	miR-141	Increase the genetation of CXCL1 and recruit Tregs into MPE	+	[60]
		/	CCL22	Exert inhibitory function	+	[17, 64]
		/	Latency‐associated Peptide(LAP)	CD39^+^Tregs inhibit Th17 proliferation and differentiation	+	[65]
		/	IRF4	Promote the conversion of Tregs to Th17-like T cells	–	[67]
		/	IL-8	Increase MMP-2/9 activity and tumor metastatic activity	+	[68, 69]
B cells		IL-10, TGF-β	PD-1/PD-L1	Activated naive B cells suppress the expansion of Th17 cells	+	[75]
TAMs	M1,M2	IL-1, IL-6, IL-12, TNF-α, IL-10, PGE2, TGF-β, MMPs, VEGF	IL-8	Up-regulate TGF-β and increase CCL22	+	[68]
			TGF-β	Reverse impaired cytotoxic effects of CD4^+^ and CD8^+^ T cells	–	[47]
			IL-10, M-CSF, TGF-β	Induce TAMs toward M2 type polarization	+	[80]
			TNF-α and IL-10	Induce PD-L1 expression, lead to T cells dysfunction	+	[81]
DCs		MHC-II	IL-10 and VEGF	Inhibit the antigen-presenting function of DCs	+	[89]
		/	TLR4/7/8 Agonist	InfDCs induce differentiation of CD4^+^ memory T cells into Th1	–	[90]
MDSCs		VEGF, bFGF, MMPs	VEGF	Increase vascular permeability and promote angiogenesis	+	[91, 94]
			IL-12, IL-10	Downregulate IL-12 expression and upregulate IL-10, suppress NKs killing activity and DCs maturation	+	[97, 98]
NKs		Perforin, Granzyme	TGF-β, IL-10, PGE2	Inhibit NKs activity	+	[103]
		/	IL-2	PE-NK cells have high killing activity, are not inhibited in TME	–	[106]
		/	IL-15	PE-NK cells control tumor growth in vivo	–	[107]
Asbestos-related macrophages		/	ROS	Generate ROS and induce apoptosis in pleural mesothelial cells	+	[110–112]
		TNF-α, IL-1β, IFN-γ	iNOS	Activate iNOS and be link to the fibroproliferative and neoplastic effects of asbestos	+	[115]
		/	HMGB1	Release of TNF-a and enhance the activity of NF-κB	+	[123, 124]

## Immune Modulation in asbestos-related MPE-TME

Asbestosis is caused by long-term inhalation of asbestos dust, which leads to the proliferation of connective tissue in lung interstitium and pleura, and is susceptible to lung cancer, malignant pleural or peritoneal mesothelioma and other malignant tumors, of which 15–20% are asbestosis with lung cancer. The main target of asbestos fibers is thought to be lung epithelial cells and pleural mesothelial cells in regard to carcinogenesis. Asbestos-related cancers can invade anywhere in the lungs and can arise from either pleural layer. Aggregated asbestos fibers cannot be “digested” by phagocytes which instead results the numbers of reactive multinucleated giant cells increased. Uncontrolled proliferation leads to Mesothelium mutation and carcinogenesis. After asbestos enters the alveoli, a large number of alveolar macrophages “swim out”, and phagocytes generate substantial amounts of reactive oxygen species (ROS) upon phagocytosis [110]. Brian et al. [111] observed that intracellular oxidative burst as rat lung-derived macrophages (NR-8383) phagocytize erionite fibers. Asbestos induces apoptosis in pleural mesothelial cells relied on ROS, which is also likely to allow the abnormal survival of mesothelial cells with asbestos-induced mutations via escaping from this pathway [112]. Some studies showed that the addition of either crocidolite or chrysotile fibers to cytokine-stimulated cell cultures upregulated the generation of nitric oxide (NO) by rat alveolar macrophage [113] as well as by rat parietal pleural mesothelial cells [114]. Shogo and colleagues demonstrated that asbestos inhalation can induce inducible nitric oxide synthase (iNOS) activation and peroxynitrite formation in *vivo* [115]. Moreover, chrysotile and amosite inhalation can induce proliferative lesions in rat airway and proximal alveolar epithelium [116, 117]. Asbestos exposure is characterized by protracted macrophage accumulations at the sites of asbestos fiber deposition [118, 119], and upregulated secretion of cytokines, TNF-α, IL-1β, and IFN-γ, which induce iNOS activation [120–122]. Thus, the current findings of iNOS activation and nitrotyrosine formation are likely to be closely associated with asbestos-induced pleuro-pulmonary cytokine activation. It is conceivable that activation of iNOS within alveolar and pleural macrophages may be linked to the fibroproliferative and neoplastic effects of asbestos. Yang’s team [123] found that upon asbestos exposure, high mobility group box-1 (HMGB1) is released by reactive macrophages and other inflammatory cells, as well as by human mesothelial cells (HMC). HMGB1 is a critical regulator in the initiation of asbestos-mediated inflammation leading to the release of TNF-a and subsequent NF-kB signaling [123]. And HMGB1 can promote tumor formation, progression, and metastasis through enhancing the activity of NF-κB [124]. However, impared T-cell function is not necessarily a common finding along with asbestos-associated malignancy [125].

## The role of immune microenvironment cells in MPE immunotherapy

The natural immune response relies on the interaction between the adaptive and the innate immune systems. In anti-tumour immunity, the basic purpose of this response is to monitor, detect, and destroy tumour cells [126]. Current treatments for MPE (such as palliative care, intrathoracic chemotherapy, and radiotherapy) have certain limitations. Modern immunotherapy has significantly improved both the efficacy and the possibility of personalized treatment for MPE. Both monotherapy and combined regimens are likely to be further studied in the coming years.

### T lymphocytes

T lymphocytes efficiently navigate and scan almost all parts of the body for unwanted or foreign substances; thus, naive and effector T cells are highly skilled migrants that are critical for immune surveillance and the development of adaptive immunity against both infection and cancer.

#### CTL cells

In principle, CTLs could target cancer through one of two pathways, both of which are based on active or passive immunity. CD8^+^ T cells exhibit important anti-tumour properties in the immune response [127]. Studies have shown that patients with MPE have defects in the recruitment of CD8^+^ T cells into the pleura [128], which reduces the cytotoxic activity of CTLs. One experimental study found that CD8^+^ T cells in the MPE could both induce apoptosis and produce IFN-γ after being cultured *in vitro* for 24h; subsequently, immunosuppressive MDSCs and soluble lactic acid and lactate dehydrogenase were found to be potential factors for T cell dysfunction, providing an important reference value for understanding the potential efficacy of T-cell targeted therapy in MPE patients [35]. CD8^+^ T cells in the MPE can mediate partial cytotoxicity against autologous non-CD45^+^ tumour containing cells after only 24 h of culture, representing a potential avenue for obtaining tumour-specific T-cell subsets in a minimally invasive manner that may be a useful screening method by which patients that are likely to respond to T-cell-targeted immunotherapy can be identified [35]. The intrapleural administration of IL-2 is a representative method for the treatment of MPE [129]. IL-2 treatment can reverse the exhaustion of CD8^+^ T cells in MPE [40], prolonging the survival period of patients [130], and increase the number of CD3^+^ T cells and NK cells, enhancing the immune response and reducing the incidence of MPE [131]. The intrapleural injection of IL-2 has been reported effective in reducing tumour-associated malignant pleurisy [132]; the level of CEA decreased in MPE patients treated with IL-2, suggesting that the MPE malignancy was reduced after treatment. Targeting T cell depletion (including PD-1) may be an effective therapeutic strategy with which MPE can be controlled in lung cancer patients [40]^.^ The critical role of CD4^+^ T cells in driving anti-tumour immunity has been widely recognized in recent years. In clinical studies, Catherine Wu et al. found that a personalized neoantigen vaccine for melanoma patients induced a tumour-specific response, mainly in CD4^+^ T cells [133]. The anti-tumour CD4^+^ T cells in colorectal cancer patients receiving oxaliplatin chemotherapy response can still be observed in some patients three months after treatment [134]. Several studies have also found that the synergistic effect of CD4^+^ T cells and CD8^+^ T cells in the anti-tumour immune response extends to CAR-T cell adoptive immunotherapy [53]^.^ Although it is clear that CD4^+^ T cells provide critical assistance for the anti-tumour immune response, the antigen repertoire that is recognized by CD4^+^ T cells in the TME remains relatively unexplored. Both deeper understanding and the rational targeting of autologous CD4^+^ T cells are required to maintain a durable and powerful anti-tumour response when using CD4^+^ T cell immunotherapy for MPE if clinical benefits are to be achieved.

#### Adoptive immunotherapy

Adoptive cell therapy (ACT) is a process in which tumour-reactive lymphocytes are harvested from a patient, cultured *in vitro*, and then infused back into the patient as therapy. In this way, reactive T cells are able to eradicate tumours by recognizing and targeting tumour-associated antigens (TAA), resulting in a strong tumour immune response [135]. ACT is a promising immunotherapy, and the most mature clinical treatment method, chimeric antigen receptor (CAR) T cell therapy, is achieving encouraging results. CARs can bind directly to lipids, proteins, and carbohydrates, expanding the range of cell surface targets [136]. The first generation of CARs only included the CD3ζ signaling domain, which was fused with extracellular single-chain antibodies to modify and activate T cells [137]. However, the short survival time of these cells meant that they could not effectively activate T cells. Second- and third-generation CARs possess costimulatory molecules (e.g., CD28, 41BB) that increase the cell persistence and killing capacity [138, 139]. The current, fourth-generation CARs contain nuclear factor-activated T cells (NFATs) and can induce large numbers of cytokines (IL-12, IL-15, granulocyte-macrophage colony-stimulating factor) to regulate the TME [140]. CAR-TCRs are activated to target the tumour-specific antigens (TSAs) that are expressed on the surfaces of cells. CD19, which has been found expressed on most B cells, has been selected as a CAR target [141]. CAR-T cells have achieved great success in the treatment of acute and chronic lymphocytic leukemia, re-invigorating exploration into the field of solid tumour immunotherapy [142, 143]. The FDA has approved two CD19 CAR-T cell antibodies for the treatment of hematological B-cell malignancies [144, 145], and recent studies have suggested that CAR-T cell therapy is also a promising strategy for the treatment of NSCLC and MPM [135]. Candidate target antigens currently being studied in lung cancer and MPM clinical trials include overexpressed TAAs (Carcinoembryonic antigen, CEA; Disialoganglioside, GD2; Glypican-3, GPC3; Human epidermal growth factor 2, HER2; Mesothelin, MSLN; PD-1; Receptor tyrosine kinase-like orphan receptor, ROR1), novel gene products (Epidermal growth factor receptor, EGFR), the aberrant glycosylation (Mucin1, MUC1) that results from splice variants, and interstitial factors associated with the TME (Fibroblast activation protein, FAP; Vascular endothelial growth factor receptor 2, VEGFR2) [135].

CEA is an antigen that is expressed during fetal growth and development, but its expression decreases after birth and is not expressed in normal adult tissues and differentiated cells [135]. The expression of CEA is increased during the occurrence and development of lung cancer [146], and it has also become a valuable tumour marker for monitoring the treatment response, providing a basis for establishing phase I clinical trials that can evaluate the efficacy and safety of CEA-targeted CAR-T cells in the treatment of lung cancer (NCT02349724 and NCT04348643).

GPC3 is a heparan sulfate proteoglycan (HSPG) that plays an important role in the growth, differentiation, and migration of cells [147]. It is highly expressed in lung cancer cells and is barely expressed in non-tumour cells [148], indicating that it is an attractive target for CAR-T cell therapy. Li et al. [148] developed third-generation GPC3-targeted CAR T cells (CARgpc3) and found that the cells exhibited a highly activated state, specifically in lysing tumour cells against GPC3- expressing lung squamous cell carcinoma (LSCC) cells and producing a large number of cytokines (IL-2, IL-4, IL-10, IFN-γ, TNF-α) that suggest T cell activation. CARgpc3 T cells may therefore be a reasonable target for immunotherapy in patients with LSCC.

HER2 is a member of the ErbB receptor tyrosine kinase family that is involved in cell proliferation and angiogenesis [149] and plays an important role in the pathogenesis of various cancers [150]. The PanErbB inhibitor Afatinib has been reported an effective drug for the treatment of HER2 positive NSCLC [151], suggesting that HER 2 may be a target of CAR T cells. In one phase I/II study, the three-day infusion of anti-HER2 CAR-T cells in patients presented no unacceptable toxicity (NCT01935843). Another study has been withdrawn due to safety concerns (NCT02713984). So far, no clinical studies have investigated the use of anti-HER2 CAR-T cell therapies for NSCLC. In one animal experiment, HER2 CAR T cell therapy inhibited tumour growth but did not eliminate it completely, in either orthotopic or subcutaneous NSCLC mouse models [152, 153].

MSLN is a cell surface glycoprotein that is overexpressed in epithelial tumours (MPM and NSCLC). MSLN is considered to be a promising target because it is overexpressed in 69 % of lung adenocarcinomas [154] and promotes the development and metastasis of lung cancer cells [155]. Several studies are currently evaluating MSLN-CAR-T cell delivery approaches, including traditional systemic intravenous administration and local intrapleural administration (NCT01583686, NCT02414269, NCT02580747, NCT02159716, NCT01355965). Adusumilli et al. observed that the intrathoracic injection of CAR T cells was more effective than the systematic infusion of T cells [155].

PD-L1 has made substantial progress in the treatment of NSCLC, and PD-L1-targeted CAR-T cells have strong cytotoxic effects against NSCLC both in vivo and in vitro [156, 157]. Liu et al. [156] constructed a CAR-T cell targeting PD-L1 by using single-chain antibodies against human PD-L1, the CD8 hinge, TM domain, CD3ζ signaling domain, and the 4-1BB co-stimulatory domain. The cytotoxic effect of CAR-T cells on PD-L1-low NSCLC cells was found to be enhanced with subtherapeutic doses of local radiotherapy, resulting in lower tumour cell proliferation rates than those obtained with monotherapies [156]. In addition, CD4^+^ and CD8^+^ PD-L1-CAR-T cells exhibited strong cytotoxicity when targeting PD-L1-high tumour cells [156]. These results suggest that PD-L1-CAR-T cells can maintain anti-tumour activity.

ROR1 is a key carcinoembryonic glycoprotein that maintains the balance of pro-survival and pro-apoptotic signaling in lung adenocarcinoma [158, 159]. Wallstabe et al. [160] developed a microphysiological three-dimensional (3D) model of lung and breast cancer that provides better characterization of the solid tumours that are encountered by CAR-T cells. The system was used to evaluate the anti-tumour effect of ROR1CAR-T cells composed of a ROR-1-specific single-chain antibody, an IgG4-Fc-derived hinge, the CD28TM domain, and 4-1BB-CD3ζ or CD28-CD3ζ signaling modules [160]. The results showed that ROR1-CAR-T cells had strong anti-tumour activity within 3 d in a dose-dependent manner [160]. A considerable percentage of the tumour cells were eradicated following injection with even the lowest dose of CAR-T cells. In vitro and in vivo NSCLC models, ROR1-CAR T cells maintained their anti-tumour activity, cytokine secretion, and proliferation [160, 161].

EGFR belongs to the family of HER/ErbB receptor tyrosine kinases that transduce extracellular growth signals into cells [162] and are amplified or mutated in a variety of cancers [163]. In vitro experiments found that EGFR-CAR T cells exhibited strong cytotoxic activity [164]. In a phase I study (NCT01869166) conducted at the Chinese People’s Liberation Army Hospital, the infusion of escalating doses of EGFR-CAR-T cell was tested in 11 patients with advanced relapsed NSCLC; EGFR-targeted CAR-T cell infusions were well tolerated by patients and pathologically EGFR-positive tumour cells were eradicated, with two patients achieving PR, and five patients reaching disease stability [165]. In an ongoing phase I clinical trial at Sun Yat-Sen University, CXCR-5-modified anti-EGFR CAR-T cells are being used to treat patients with NSCLC and are being evaluated for efficacy and safety (NCT04153799). Of the 11 evaluated patients who received three different doses, two had a partial response and five attained disease stability over 8 months. Therefore, anti-EGFR CAR-T cells may be a feasible means of treating EGFR-positive NSCLC patients, although more clinical studies are needed to confirm these results. EGFR mutations can be detected in MPE, but the response rate of EGFR tyrosine kinase inhibitors is lower in MPE than solid tumours, and no relevant reports have been produced detailing the use of EFGR CAR-T for the treatment of MPE.

MUC1 is a transmembrane glycoprotein that is overexpressed in many cancers, including NSCLC. MUC1 and PSCA CAR-T cells have been found to show independent and synergistic anti-tumour effects in a patient-derived xenograft model of NSCLC [166]. In the ongoing phase I/II clinical trial conducted by the PersonGen Biotherapeutics (Suzhou) Co., Ltd., anti-MUC1 CAR-T cells are being used to treat advanced refractory solid tumours, including NSCLC (NCT02587689). Another phase I/II clinical study evaluating the safety and efficacy of the combination of anti-MUC1 CAR-T cells and PD-1 knockout in patients with advanced NSCLC (NCT03525782) is also underway. The First People’s Hospital of Hefei, China, is about to complete a phase I/II study of MUC1-CAR T cells in patients with MUC1^+^ advanced refractory NSCLC (NCT02587689), and is close to completing a similar ongoing study in which MUC1-CAR-pNK cells (with CAR structures implanted into placenta-derived NK cells) are applied in patients with advanced refractory NSCLC (NCT02839954). Additionally, Tmunity Therapeutics recently registered a phase I trial to test the safety, tolerability, feasibility, and preliminary efficacy of MUC1 targeted CAR-T cells (TnMUC1) in TnMUC1-positive advanced cancers, including NSCLC (NCT04025216). In patient-derived xenotransplantation models, anti-MUC1 CAR-T cells have not been found to significantly inhibit the growth of NSCLC tumours [166].

FAP is an integral membrane gelatinase that controls both fibroblast growth and the epithelial to mesenchymal transition in most human malignancies [167]. Both FAP molecules themselves and FAP-positive cells in the TME may be involved in the proliferation, invasion, angiogenesis, and extracellular matrix (ECM) remodeling of cancer cells [168]. The University of Zurich is currently enrolling patients with MM MPE for phase I single-dose FAP CAR-T cell therapy (NCT01722149). The topical application of anti-FAP CAR-T cells, combined with CD28, ΔCD28, 4-1BB co-stimulatory domains and PD-1 inhibition has been shown to provide transient tumour control and improve the survival in humanized mouse MM models. At the same time, disease stability has been attained for one year in MM patients with MPE [169]. FAP should therefore be further evaluated as a specific antigen for CAR-T therapy.

#### Immune checkpoints

Immune checkpoint molecules are inhibitory receptors on the surface of immune cells that ensure proper regulation of the immune response. It is well known that inhibitory molecules on the surface of T cells or tumour cells can promote T cell dysfunction and exhaustion, thereby suppressing the anti-tumour immune response. These co-suppressive immune checkpoint molecules include programmed death-1 (PD-1), programmed death ligand 1 (PD-L1), cytotoxic T lymphocyte-associated antigen-4 (CTLA-4), T cell immunity globulin and mucin domain containing protein 3 (TIM3), lymphocyte activation gene 3 (LAG3), and T-cell immunoglobulin and ITIM domain (TIGIT) receptor [170].

##### CTLA-4

Cytotoxic T lymphocyte-associated antigen 4 (CTLA-4) is considered a gatekeeper immune checkpoint. CTLA-4 is an inhibitory receptor that downregulates the initial phase of T cell activation [171]. When CTLA-4 is mobilized from the cytoplasm and into T cells, it binds to receptors CD80 and CD86 on APC, mediating direct inhibition of the MHC-TCR pathway and reducing the T cell effector function [172]. CD28 and CTLA-4 are two important T-cell co-stimulatory receptors that share the same ligands; however, CTLA4 has a much higher (20-fold) affinity for the ligands, thus outperforming CD28 [126]. After CD8^+^ T cell activation, the expression of CTLA-4 receptor is up-regulated, transmitting inhibitory signals down to balance the input and ensure the controlled activation of the CD8^+^ T cells. CTLA-4 activation interferes with the motility of CD8^+^ T cells and the formation of stable binding with APC, thereby reducing the contact time between cells [173]. Ipilimumab is as yet the only FDA-approved anti-CTLA-4 anti-tumour drug [174]. In studies of stage IIIB/IV NSCLC, ipilimumab plus paclitaxel/carboplatin did not prolong PFS or OS [175].

##### PD-1/PD-L1

Programmed cell death protein 1 (PD-1) is an important immune binding site receptor. PD-L1 and PD-L2 are PD-1 ligands and are overexpressed on the surface of tumour cells. PD-L1 is expressed in tumour cells and the antigen-presenting cells of tumours to varying degrees, with a strong inhibitory effect in the TME [176]. PD-1 mediates the immunosuppressive effect by binding to its ligands. When PD-1 binds to ligands PD-L1 (B7-H1) and PD-L2 (B7-DC), it specifically moves TCRs out of the so-called central supramolecular activation cluster (CSMAC), reducing T cell survival and inhibiting T cell proliferation and the IFN- γ, TNF-α, and IL-2 production [177]. In addition, the involvement of PD-1 shifts the metabolism of T cells from glycolysis to fatty acid β-oxidation, leading to the accumulation of reactive oxygen species, mitochondrial damage, and apoptosis [126, 178]. The interaction of PD-L1-PD-L2-PD-1 antagonizes CD80-CD28 co-stimulation and strongly counteracts TCR signaling, resulting in a decreased cytokine production by CD8^+^ T cells and even T cell depletion [179]; TCR signaling can be enhanced by blocking PD-1 with antibodies, thereby restoring T cell function. Currently, the U.S. Food and Drug Administration (FDA) and/or the European Medicines Agency (EMA) have approved two PD-1 antibodies (nivolumab and pembrolizumab) and two PD-L1 antibodies (atezolizumab and durvalumab) for the treatment of NSCLC [180–182]. Pembrolizumab (Keytruda) and nivolumab (Opdivo) are human IgG4 PD-1 immune checkpoint suppressive antibodies [183] that interfere with PD-1 mediated signal transduction and have the ability to reverse T cell functional inhibition, restoring anti-tumour immunity [184]. Atezolizumab (Tecentrip) and durvalumab are humanized engineered IgG1 monoclonal antibodies against PD-L1, which can inhibit PD-L1 binding to PD-1 or B7-1, thereby further enhancing the immune response to cancer cells [185].

A study of various treatments on patients with advanced NSCLC with PD-L1 expression for at least 1 % of tumour cells has found that pembrolizumab treatment extended the OS compared to conventional docetaxel chemotherapy (12.7 vs 8.5 months) [186]; another study showed that in patients with advanced NSCLC and high PD-L1 expression on at least 50 % of tumour cells, median progression-free survival, overall survival, and response rates were significantly higher in patients receiving pembrolizumab than in those receiving docetaxel [187]. In the advanced NSCLC trial, patients treated with nivolumab had a higher response rate (20 % vs 9 %) than those treated with docetaxel, with significantly longer PFS (3.5 vs 2.8 months) and 5-year OS (13 % vs 3 %) [188, 189].

In treatment-naïve patients with metastatic NSCLC, Atezolizumab increased median OS by 7.1 months (20.2 vs 13.1 months) and reduced the incidence of grade III or IV adverse events (30.1 % vs 52.5 %) as compared to platinum-based chemotherapy [190]. The placebo-controlled Phase III PACIFIC trial of patients with Stage III NSCLC demonstrated that durvalumab is an effective consolidation therapy, inducing significant improvement in median PFS (16.8 vs 5.6 months) [191]. Analysis of the latest study using randomized patients in the PACIFIC Trial showed that 49.6 % were still alive after 4 year. The median OS of 4 year after initial treatment (47.5 vs 29.1 months), 4-year OS rate (49.6 % vs 36.3 %), and 4-year PFS rate (35.3 % vs 19.5 %) all indicate that durvalumab benefits clinical patients on a continual basis [192].

In fact, studies have found limited benefits from taking checkpoint inhibitor drugs [193], and side effects have been observed in the digestive system, skin, endocrine, and liver, alongside abnormal immune reactions [194]. Efforts are underway to reduce the toxicity of these drugs and increase their activity by co-administration with one or more therapies, as demonstrated by studies in which PD-1/PD-L1 inhibitors are combined with CTLA-4 inhibitors. For example, the effective rate of nivolumab plus ipilimumab in the treatment of advanced NSCLC is higher than that of monotherapy. A trial study found that median survival (17.1 vs 13.9 months), 2-year OS (40 % vs 32.8 %), and related adverse events (32.8 % vs 36 %) were significantly improved in such patients as compared with chemotherapy [195]. The latest study randomized 605 MPM patients for the administration of combined immunotherapy with nivolumab and ipilimumab versus chemotherapy. Comparing the combined use of nivolumab and ipilimumab with platinum and pemetrexed chemotherapy produced a median survival of 18.1 versus 14.1 months, a 2-year OS rate of 41 % versus 27 %, and a significantly improved OS rate, suggesting clinical significance [196]. The FDA approved nivolumab + ipilimumab for the treatment of unresectable MPM in October 2020 [196].

#### Tregs

Tregs, a subgroup of CD4^+^ T cells, are powerful immunosuppressive cells that inhibit anti-tumour immunity by inhibiting effector T cell (Teffs) activity. They contribute to the development of an immunosuppressive TME, thereby promoting immune escape and cancer progression [197, 198]. Tregs are induced into the pleural cavity by the chemokine CCL22 [17]. Since Tregs have an inhibitory effect on peripheral effector T cells, the removal of local Tregs may be key to treatment for MPE. The percentage of CCR4^+^CD4^+^ T cells has been found higher in MPE patients than non-MPE patients [199], and the Tregs in the peripheral blood strongly expressed CCR4 compared with effector T cells [17]. Anti-CCR4 mAb may be an ideal treatment approach for patients with CCR4 positive tumours [200]. Recent studies have found that the CCR4 antagonist can enhance anti-tumour immune activity by reducing Tregs aggregation in the TME, and the combination of CCR4 inhibitor and CTLA-4 has a more significant effect [201]. This could serve as a novel strategy for the treatment of MPE by overcoming the inhibitory effect of CCR4^+^ Tregs. Tumour cell-derived IL-1α is a major inducer of Tregs that attracts the chemokine CCL22, and therapeutic blockade of the IL-1 pathway may also be an effective approach to limiting tumour-induced immunosuppression [202]. Type I interferon can block the chemokine CCL22 that attracts Tregs, thus helping to limit Treg recruitment by tumours and inhibit tumour progression [203]. Ye et al. [70] showed that TNF-TNFR2 interaction may have an important impact on the activation and expansion of Tregs in MPE. After blocking TNFR2, the anti-tumour activity of CD8^+^ CTL was significantly enhanced. Targeting TNFR2 thus offers a new possibility of selectively eliminating MPE Tregs with little effect on normal Tregs, allowing immune homeostasis to be maintained. However, targeting TNFR2^+^ Tregs may reduce the likelihood of the development of systemic autoimmune inflammatory responses, which can cause significant collateral damage to normal tissues after cancer immunotherapy [204]. Further research is thus required to provide more effective treatments.

### TAMs

TAMs can be divided into the typical activated M1 and the alternate activated M2 subtypes. M1 is characterized by the secretion of a variety of pro-inflammatory cytokines, while many of the cytokines that are produced by M2 are beneficial for tumour progression [205]. Yang et al. [206] found that Pseudomonas aeruginosa mannose-sensitive hemagglutinin (PA-MSHA) re-cultured CD163^+^ TAMs into M1 macrophages through the TLR4-mediated pathway. Clinically, PA-MSHA has been used to treat the MPE that is associated with lung cancer [207]. Within 12 h of PA-MSHA treatment, the amount of pleural effusion gradually decreased, and the cytotoxicity of CD163^+^ TAM-injured NK cells was restored [206]. The inhibition of TAMs or promoting the polarization of M2 to M1 are thus effective therapeutic strategies that can inhibit MPE. The TGF-β that is secreted by TAMs is most strongly expressed in cancer, especially malignant exudates, and studies have shown that TGF-β may be associated with lung cancer progression in MPE patients [207]. Monoclonal antibodies, vaccines, antisense oligonucleotides, and small molecule inhibitors can be used to block TGF signaling. One study has also shown that TGF-β facilitates immune escape in tumours. Anti-TGF-beta therapy has been used to reverse TGF-β-mediated immunosuppression and produced significant clinical efficacy in cancer patients [208]. Inhibition of the TGF-β-mediated signaling pathway is likely to effectively control MPE tumour cell proliferation, providing a new approach to MPE immunotherapy [209]. TAMs produce the chemokine CCL22, which recruits Tregs to the TME and reduces anti-tumour immunity [210]. Fucosan can inhibit the tumour cell migration that is induced by the M2 macrophage conditioned medium, the function of CD4^+^ T lymphocytes, and especially the recruitment of Tregs by inhibiting CCL22 via the activity of NF-κB, which reduces CCL22 production. Therefore, it can be used as an anti-tumour drug that targets macrophages, and may provide a new approach to tumour immunotherapy in the future [211]. Further studies by Wang et al. [68] found that IL-8 induces TGF-β up-regulation in TAMs, which mediates the production of CCL22 and leads to the formation of immunosuppressive TME in MPE. Blocking IL-8 signaling may also be a potential therapeutic target for MPE patients.

### DCs

DCs are the most powerful antigen precursor cells that are responsible for initiating both innate and acquired immune responses. DCs play a role in initiating the anti-tumour immune response by presenting tumour-derived antigens to the T cells in tumours, and have thus become a target for tumour therapy. DC-based MPE immunotherapies have been developed by modulating the function of DCs. Di et al. [212] used IL-4, GM-CSF, and TNF-α to induce autologous peripheral blood CD34^+^ stem cells that can differentiate into DCs, and treated malignant effusion by injecting these cells back into the body cavity with a total effective rate of 54 %, a median remission time of 20 weeks, and no serious adverse reactions. In another study, DCs were pulsed with tumour cell lysates to prepare a DC vaccine that could be administered intradermally or intravenously. The vaccine was well tolerated and was able to induce an immune response in the tumour cells of patients with MPM [213]. Later, it was found that the combination of low-dose cyclophosphamide and DCs in MPM can reduce the number of Tregs, and the disease of 8 out of 10 patients was controlled. Seven patients had a survival rate of more than 24 months. DC combined with CTX treatment was safe and feasible, with no obvious adverse reactions [214]. More clinical trials using this method may thus provide more therapeutic approaches for the treatment of MPM-derived MPE. He et al. [215] treated MPE and MA with an intrapleural infusion of mixed DC-cell-induced killer cells (CIK), the total effective rate within 4 weeks was 38 %, and the clinical control rate was 70.3 %. Local treatment with DC-CIK did not improve the patient’s condition; however, the generation of pleural effusion and ascites was controlled and the related symptoms were relieved. They also found that DC-CIK intracavity infusion did not achieve a higher control rate when combined with chemotherapy, indicating that DC-CIK infusion may be an effective treatment for controlling the occurrence and development of MPE when used alone [215]. The clinical application of DC vaccines is a new focus for research. The use of DC vaccines is a therapeutic method in which antigen-loaded DCs are stimulated by anti-inflammatory cytokines and delivered to promote the anti-tumour immune responses of tumour antigen-specific CD8^+^ T cells [89]. The toxicity of DC vaccines is much lower than that of other tumour therapies; however, factors such as immunosuppressive TME and the immune escape of tumour cells can reduce the efficacy of DC vaccines. Since ICB therapy can reverse the immunosuppressive microenvironment, combining ICB with DC vaccines has been found to activate CD8^+^ T cells *in vivo* and can be synergized with PD-1 blockers to exert anti-tumour immunity [216]. Restoring the activity of DCs has considerable potential in advancing tumour immunotherapy, and future studies targeting the activity of DCs are critical to advancing MPE therapy.

### MDSCs

MDSCs are the main immunosuppressive cells that maintain tumour progression in the TME and are a major obstacle to immunotherapy. Targeting MDSCs is thus an effective measure that can improve immunotherapy interventions. MDSCs can currently be targeted by depleting circulating and tumour-infiltrating MDSCs *in vivo*, preventing the recruitment and transport of MDSCs, inhibiting the immunosuppressive function of MDSCs, and differentiating MDSCs into a non-suppressive immune state [217]. Qin et al. [218] developed a novel therapeutic polypeptide-FC fusion protein that targets the S100A family proteins and selectively depletes MDSCs without acting on other pro-inflammatory immune cells. Several studies have also found that CCR5 blockage can inhibit the recruitment and immunosuppressive activity of MDSCs [219]. For example, CCR5 antagonists can inhibit the metastatic potential of basal breast cancer and tumour growth [220]. Elevated levels of CCL2 and CCL5 in the TME recruit MDSC through the chemokine receptor [221, 222] CXCR2. CXCR2^+^ MDSCs promote tumour expansion, metastasis, EMT and T-cell depletion in breast cancer [223]. Therefore, they may have a certain inhibitory effect on the MPE that is associated with breast cancer, providing certain reference significance for future studies investigating the targeting of MDSC immunotherapy to MPE. CSF-1R is a tyrosine kinase receptor that, when bound to the ligand CSF-1, promotes the differentiation and expansion of MDSCs into MDSCs and TAMs, in addition to promoting the migration of MDSCs to tumours [224]. Research has indicated that CSF-1R inhibition and CXCR2 antagonism could reduce the number of TAMs and PMN-MDSCs and improve the efficacy of anti-PD-1 [225]. This may also reduce the immunosuppressive effect of MPE and enhance the killing effect of other immune cells, thus achieving the goals of such treatment. Phosphodiesterase-5 (PDE-5) inhibitors can also eliminate MDSC immunosuppression mechanisms by targeting MDSC expression and the function of ARG1 and iNOS [226]. The administration of PDE-5 inhibitors (such as sildenafil and tadalafil) has been reported to reduce the inflammatory response in the TME, replaying the anti-tumour immune rejection effect and prolonging the survival time *in vivo* through T and NK cell activity [227, 228]. Paclitaxel has also been reported to reduce the number of MDSCs by promoting the differentiation of MDSCs into DCs in a TLR4-independent manner [229], providing a new direction for the future treatment of MPE. At present, the mechanism of MDSC immunotherapy for MPE is still relatively unknown, and in-depth research on targeted therapies for MDSCs in MPE remains necessary.

### NK cells

Tumour-associated NK cells can migrate to tumour sites *in vivo* and exert anti-tumour activity [230]. Adoptive immunotherapy using NK cells in combination with cytokines including IL-2 (it mediates NK cell activation and proliferation) is an important treatment for patients with different tumours or leukemia [231]. Studies have shown that NK cells that are derived from MPE have strong cytosolic activity, not only on allogeneic tumour cells, but also on autologous tumour cells [106]; the interaction of MPE-derived NK cells with tumour cell-associated suppressor or regulatory cells may be restrained, enabling the further maintenance of efficient cytolytic function [232]. Vacca et al. [105] reported that since tumour-derived suppressors and cytokines are diluted in the effusion, they are not sufficient to induce an inhibitory effect and the effect of intercellular production is also limited, protecting the function of MPE-derived NK cells from being impaired. IL-2is known to activate and enhance the function of NK cells, and the possibility of inducing or enhancing the body’s anti-tumour activity by IL-2 infusion is indicated; since the function of MPE-NK cells is not damaged, it may be more effective to transport NK cells into the pleural cavity or inject them directly into the pleural cavity. Other studies have also shown that intrapleural injection of IL-2 induces anti-tumour activity in patients with MPM [233]. Studies involving the intrapleural injection of IL-2 have indicated that the effusion in patients with lymphoma-related MPE has disappeared, with no effusion observed for over two years [234]. However, several clinical trials have confirmed that IL-2 has toxic effects [235–237]. IL-15 antagonizes the inhibitory effect of TME and restores the killing ability of NK cells; however, the corresponding toxicity observed for IL-2 does not occur [107], providing clues for the clinical adoptive immunotherapy of MPE.

**Fig. 3:**
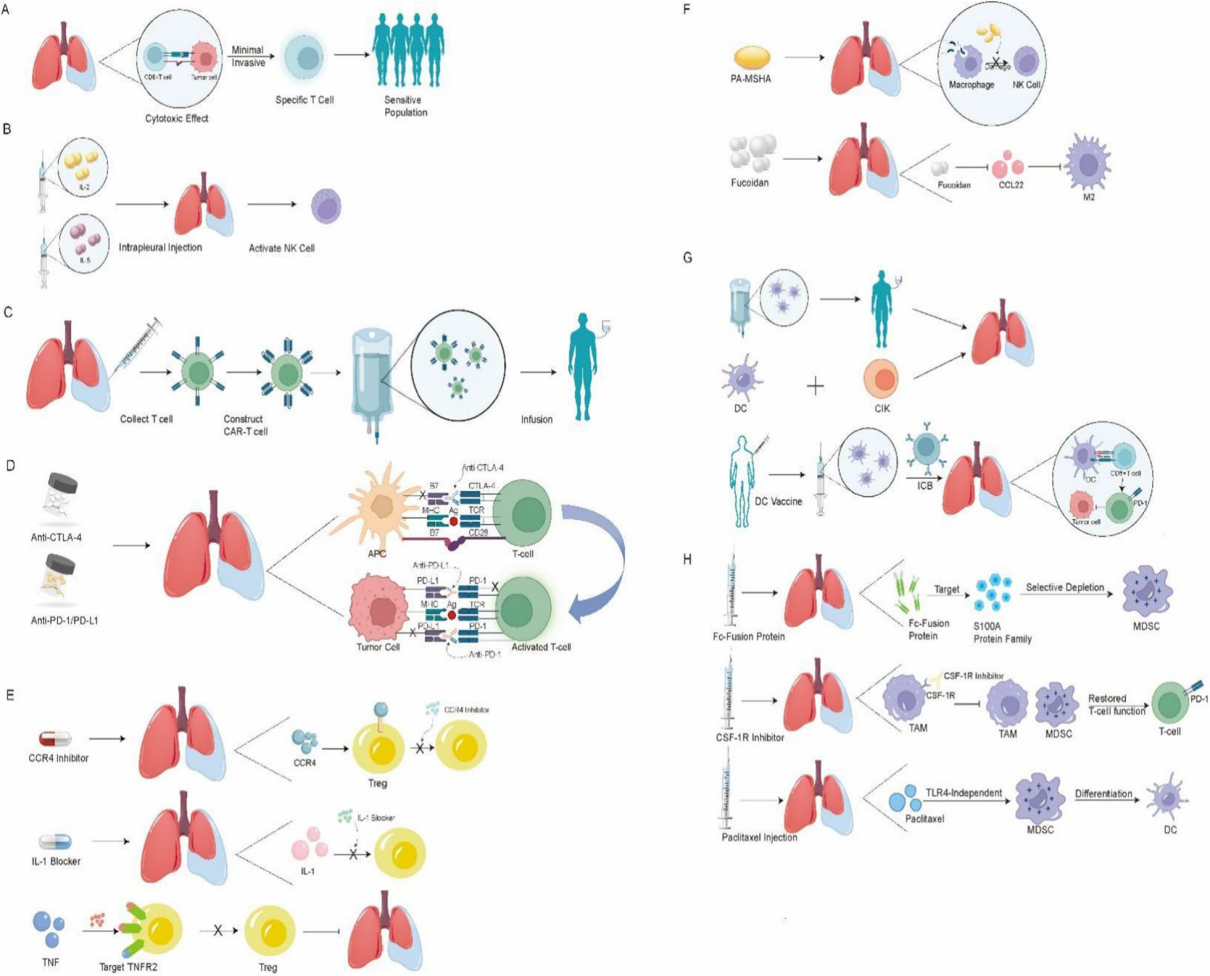
The cellular and molecular mechanisms of different immunotherapeutic approaches

The different types of immunotherapy drugs and approaches that have been clinically used or have potential value for the treatment of MPE were listed in Table 2. The cellular and molecular mechanisms of these immunotherapeutic approaches were shown in Figure 3.

**Table 2:** Immunotherapy of lung cancer with MPE

Immunotherapy	Treatment method	Target effect/target medicine	Clinically relevant effects/manifestations	References
Target T cells	Minimally invasive technologies	Specific T cells	Screen patients who are susceptible to T cell-targeted therapies	[35]
	Intrapleural administration of IL-2	CD8^+^T cells, CD3^+^T cells	Prolong patients survival, alleviate pleurisy	[129, 130, 132]
Adoptive cell therapy	CAR-T	CEA	Phase I clinical trial: evaluate the efficacy and safety of CAR-T therapy	NCT02349724 NCT04348643
		GPC3	Eliminate GPC-expressing LSCC cells and produce cytokines(IL-2,IL-10,IFN-γ,TNF-α)	[148]
		HER2	Afatinib is a treatment option for HER2 mutation-positive NSCLC	[151]
		/	Exhibit acceptable toxicity in phase I/II studies	NCT01935843
		MSLN	Inject CAR-T cells is better than systemic infusion of T cells	NCT01583686 NCT02414269
		PD-L1	Killing effect enhanced and proliferation rate of tumor cells was low	[156]
		ROR1	Microphysiological 3D models exhibited anti-tumor activity	[160]
		EGFR	Phase I trial: EGFR-CAR-T cells infusion was tested in NSCLC patients	NCT01869166 166
		/	Evaluate CXCR-5-modified anti-EGFR-CAR T cells immunotherapy	NCT04153799
		MUC1	Anti-MUC1-CAR T cells is used in an open Phase I/II clinical trial	NCT02587689
		/	Phase I/II trial: evaluate anti-MUC1 CAR-T cells with PD-1 gene knockout	NCT03525782
		/	MUC1-CAR T cells treatment in phase I/II study	NCT02587689
		/	MUC1-CAR-pNK cells study: implant CAR structure into NK cells	NCT02839954
		/	CAR-T cells target TnMUC1 in a phase I trail	NCT04025216
		FAP	Phase I clinical trial: anti-FAP-Δ-CD28/CD3ζ CAR-T cells with PD-1 inhibition improved survival	NCT01722149 [169]
ICB	CTLA-4	Ipilimumab	Added carboplatin and paclitaxel didn’t prolong NSCLC PFS or OS	[174, 175]
	PD-1/PD-L1	Pembrolizumab	Improve OS (12.7 vs 8.5 months)	[186]
		/	Improves PFS, OS and efficacy rates	[187]
		Nivolumab	Improved efficacy rates (20% vs 9%), PFS (3.5 vs 2.8 months) and 5-year OS (13% vs 3%)	[188, 189]
		Atezolizumab	Median OS prolong 7.1 months and incidence of garde 3/4 adverse events was low	[190]
		Durvalumab	Phase III Pacific trial: the median PFS(16.8 vs 5.6 months) was improved	[191]
		/	4-year median OS (47.5 vs 29.1 months), 4-year OS rate(49.6% vs 36.3%) and 4-year PFS rate (35.3% vs 19.5%) improved	[192]
		Nivolumab + Ipilimumab	Efficacy rates, median survival (17.1 vs 13.9 months), 2-year OS rate (40% vs 32.8%) improved and incidence of adverse reactions (32.8% vs 36%) decreased	[195]
Tregs	CCR4 antagonist	Tregs	CCR4 and CTLA-4 inhibitors increase efficacy greatly	[201]
	IL-1 blockers	CCL22	Inhibit tumor-induced immunosuppression by attracting CCL22	[202]
	Target TNFR2	Tregs	Antitumor activity of CD8^+^T cells was enhanced remarkablely	[70]
TAMs	PA-MSHA	NKs	Pleural effusion gradually reduced in 12 h	[206, 207]
	mAb, vaccines, antisense oligonucleotides, small molecule inhibitors	TGF-β	Reverse immune suppression using antisense gene	[208]
	Fucoidan	NK-κB	Inhibit tumor cells migration, CCL22 generation and CD4^+^T cells recruitment	[211]
DCs	DCs are reinfused to thoracic cavity	CD34^+^stem-cells	The overall response rate was 54%, the median duration of response was 20 weeks	[212]
	DC-CIK	DCs	The overall response rate was 38% and the disease control rate was 70.3%	[215]
	DC vaccine therapy combined with ICB	CD8 + T cells	Show anti-tumor effect	[216]
MDSCs	Peptide-Fc fusion protein	S100A protein family	Eliminate MDSCs	[218]
	CSF-1R	TAMs, PMN-MDSCs	Boost anti-PD-1 immunotherapy efficacy	[225]
	PDE-5 inhibitors	MDSCs	Reinvigorate anti-tumor immune responses and prolong survival in vivo	[227, 228]
	Paclitaxel	MDSCs	Promote MDSCs differentiation into DCs in MPE	[229]
NKs	Intrapleural administration of IL-2	NKs	MPE disappeared and didn’t recurrent more than 2 years	[234–237]
	IL-15	NKs	Antagonize the inhibitory effects of TME and not cause corresponding toxicity	[107]

## Conclusions and prospects

Owing to the limitations of antigen type and immunosuppressive function of tumours, many therapeutic methods have so far have failed to generate satisfactory results. A favourable tumour immune environment is a prerequisite for successful cure, and the study of the immune microenvironment provides new concepts for tumour therapy. Although there is no definite basis on which immunotherapy can replace traditional therapies in the short term, study of the tumour immune mechanism provides new directions and new choices for clinical practice. However, some problems remain to be solved, such as the limited understanding of the characteristics of TME, which largely hinders the development of new targets for immunotherapy. Different tumours elicit different responses in host immune systems. Immunotherapy that works for one tumour type or patient may not work for other tumour types or patients. The characteristics of the immune environment of each tumour need to be considered to note where the immune system fails to mount an effective anti-tumour response. The MPE is a special microenvironment, and understanding the underlying factors that affect the immune cell function in the MPE is critical to improve current immunotherapy.

## Data Availability

The main data of this study can be requested directly from the authors.
